# Molecular Determinants of Cancer Therapy Resistance to HDAC Inhibitor-Induced Autophagy

**DOI:** 10.3390/cancers12010109

**Published:** 2019-12-31

**Authors:** Maria Mrakovcic, Leopold F. Fröhlich

**Affiliations:** 1 Department of Cranio-Maxillofacial Surgery, University of Münster, 48149 Münster, Germany; leopold.froehlich@ukmuenster.de; 2 Department of Medical Microbiology, University of Münster, 48149 Münster, Germany

**Keywords:** histone deacetylase inhibitor, HDACi, drug resistance, autophagy, cell death, cancer, tumor, chemotherapy, radiotherapy

## Abstract

Histone deacetylation inhibitors (HDACi) offer high potential for future cancer therapy as they can re-establish the expression of epigenetically silenced cell death programs. HDACi-induced autophagy offers the possibility to counteract the frequently present apoptosis-resistance as well as stress conditions of cancer cells. Opposed to the function of apoptosis and necrosis however, autophagy activated in cancer cells can engage in a tumor-suppressive or tumor-promoting manner depending on mostly unclarified factors. As a physiological adaption to apoptosis resistance in early phases of tumorigenesis, autophagy seems to resume a tumorsuppressive role that confines tumor necrosis and inflammation or even induces cell death in malignant cells. During later stages of tumor development, chemotherapeutic drug-induced autophagy seems to be reprogrammed by the cancer cell to prevent its elimination and support tumor progression. Consistently, HDACi-mediated activation of autophagy seems to exert a protective function that prevents the induction of apoptotic or necrotic cell death in cancer cells. Thus, resistance to HDACi-induced cell death is often encountered in various types of cancer as well. The current review highlights the different mechanisms of HDACi-elicited autophagy and corresponding possible molecular determinants of therapeutic resistance in cancer.

## 1. Introduction: Autophagy

Autophagy is an evolutionary well conserved cellular process in eukaryotes that was established already in yeast and has developed to a highly sophisticated version in mammals [1,2,3]. The basic physiologic function of autophagy is to retrieve cellular components, such as denatured, aged, damaged, or non-required molecules and organelles. This mechanism is not only important under normal physiological conditions where it occurs at basal levels for sustaining proper homeostasis (e.g., during development and differentiation) and viability but also predominantly during times of nutrient deprivation and other stressfull settings (e.g., starvation, hypoxia, oxidative stress) [4,5,6,7]. Principally, autophagy is categorized into the classes of macroautophagy, microautophagy, and chaperone-mediated autophagy, depending on the engaged pathway [8,9,10]. Furthermore, autophagy can occur either selective or nonselective, meaning that cargo for sequestration is targeted specifically e.g., via autophagy receptors such as Sqstm1/p62 or randomly e.g., bulk cytoplasm [11]. While discovery of the molecular mechanisms governing of the non-selective starvation-induced mode of autophagy can be attributed to the Nobel prize-awarded research of Yoshinori Ohsumi, it was Daniel J. Klionsky´s group that revealed the molecular process of selective autophagy by defining the cytoplasm-to-vacuole targeting (CVT) pathway, a few years later [1,12,13]. Nevertheless, the term autophagy (from the ancient Greek “aὐsόφacος“, meaning “self-eating”) was initially forged by the Belgian cytologist and biochemist Christian De Duve in 1963 for describing intracellular vesicles that contain parts of the cytoplasm and organelles in various states of disintegration [10,14].

In general, the process of autophagy is a highly regulated process including a set of signaling cascades. Nevertheless, a complex genetic program required in cellular homeostasis or cell death is still not exhaustedly explored and might also differ between basal autophagy and stimulus-induced autophagy [1,6]. General triggering signals of autophagy are very heterogeneous and comprise for instance the shortage of nutrients, the presence of aggregated protein, reactive oxygen species (ROS) in the cell, or damaged cellular organelles [15,16,17]. Aged or damaged cellular components are degraded and retained in specifically for this process allocated autophagosomes. The event of macroautophagy, representing the best investigated form of the cellular degradation program, is therefore most often simply called autophagy and describes the controlled formation of an autophagosome. It represents a vesicle with a double-layered membrane that forms around the targeted cellular component and thereby allows its separation from the remaining cytoplasm [18]. In a transitory stage, subsequent maturation and fusion of this vesicle with lysosomes, exposes the vesicle cargo to lysosomal enzyme degradation by cathepsin and calpain proteases, and redelivers it to the cell for recycling [19]. Vesicles formed in this way are then called autophagolysosomes. Taken together, this dynamic process that includes the formation, engulfment, and lysosomal fusion of autophagosomes is commonly described by the term “autophagic flux” and is defined as a measure of autophagic degradation activity [11,20,21].

Initially, autophagosomes are formed at the phagophore assembly site and was alternatively termed pre-autophagosomal structure (PAS) [22]. Presently, it is concluded that this initial structure, that recruits various autophagy-related (ATG) proteins, is derived from the endoplasmic reticulum through the formation of the omegasome [23,24]. ATG proteins, entail a specific sequence of events in formation, growth, and closure of the autophagosome and have been summarized into five stages [25]. These involve the ATG1/ULK kinase complex during initiation, the ATG12 conjugation system during nucleation, the ATG8/LC3 conjugation/deconjugation system during elongation, the phosphatidylinositol 3-kinase complex during maturation, and the ATG9/ATG9L1 cycling system during degradation. 

## 2. The Oncogenic Role of Autophagy

As autophagy represents a physiological adaptive strategy to stressfull conditions, its disruption was found to contribute to the pathogenesis of disease, such as cancer, by promoting and accelerating tumorigenesis [17,26,27,28]. Although this cellular process has been observed for half a century, the exploration of its significance for cancer development and progression has only recen tly been initiated with the identification of master regulators, such as ATG genes. In this regard, the role of autophagy in cancer holds a high potential for future therapy and is consequently a matter of intensive investigation in laboratory and in clinical studies. However, opposed to other forms of cell death such as apoptosis, autophagy has been characterized by an ambiguous role in cancer in reply to adverse stress events as it supports either an oncosuppressive or an oncosupportive mode of response [29,30,31]. Thus, on the one hand, autophagy may help in the elimination of oncogenic factors, such as oncoproteins, but on the other hand autophagy might be activated as a prosurvival response to cancer treatment, thereby reducing therapeutic efficiency. The most often unpredictable survival- or death-promoting outcome of autophagy thereby seems to rely on the duration and dose of drugs, on the type and stage of cancer, as well as its genetic predisposition [29,32,33,34,35,36,37,38]. The later fact might prevailingly relate to the mutational effects of the most commonly altered tumor suppressor genes (such as *LKB-1*, *PTEN*, *BECLIN-1*, *TP53*, *FOXO1*, *DAPK1*) or oncogenes (such as *BCL-2 family genes*, *PI3K*, *AKT*, *mTOR*, *RAS*) that together with microRNAs represent the main regulators of autophagy [39,40]. Of note, microRNAs, belonging to the cellular epigenome, have been evidenced to modify the activity of mRNAs encoding for key autophagy-promoting proteins such as *PI3K*, *ULK1*, *Beclin-1*, *LC3B*, *ATG4* family members, *ATG5*, *ATG7*, *ATG10*, *ATG12*, or *ATG16L1* [41]. Nevertheless, further factors might be found complementing the incomplete mechanistic insights concerning autophagic signaling pathways; these will presumably contribute to the transcriptional and epigenetic regulation of the complex autophagic response, resulting in cell survival or cell death triggered by disease or pharmaceutical intervention. Thus, in addition to the relatively few experimentally validated autophagy-specific transcription factors binding to the promoters of autophagic regulatory proteins, even more transcription factor interacting binding sites are predicted by bioinformatics algorithms [30]. Additionally, selective autophagy influencing the recruitment and degradation of cell survival factors in autophagosomes such as the take-up of catalase from the cytoplasm that would induce ROS generation and induction of cell death might alter the fate of the autophagic response [42,43]. Selective autophagy is a tighly regulated process that depends on autophagy receptors such as Sqstm1/p62 and NBR1 that are controlled by posttranslational modifications and connect the mostly ubiquitin-labeled cargo to proteins of the ATG8 family. These act as adaptors that attach cargo to the inner surface of the growing phagophore.

The suppressive or supportive mode of autophagy was tightlyconnected to a function of time and differentiation during tumor development. Thus, during initial phases of tumorigenesis the protective function of autophagy prevails by removing damaging agents from the cell thereby decreasing the tendency of damaged cells to transform into tumor cells. As an example, autophagy was documented to prevent the increased effects of oxidative stress by clearing damaged organelles at the cellular level [44]. Supportive models for this tumor-suppressive action of autophagy are found in hemizygous Beclin-1-deficient mice that lose their autophagic regulatory potential thereby being increasingly susceptible to tumor formation [28,45]. During later phases of tumorigenesis however, autophagy seems to be reprogrammed by the tumor cell to prevent its eradication and even support tumor progression and metastasis. For example, autophagy can help to reduce ROS-induced radical formation effects of metabolic stress products that would harm the tumor cell and supply it with nutrients thereby enhancing tumor survival [46]. These pathological circumstances however offer the possibility to expedite and overstress the autophagic program by pharmacological interference via unknown mechanisms and direct the tumor-promoting conditions towards induction of cell death. Prolonged autophagy thereby seems to deplete critical survival factors or eliminate essential cellular contents and organelles which could also promote cell death by activating apoptosis or necroptosis. Particularly, as a physiological adaption to apoptosis resistance in tumor cells, autophagy resumes a tumorsuppressive role, that confines inflammation and tumor necrosis [47,48]. This could be verified in our own model of the apoptosis-resistant uterine sarcoma cell line ESS-1; in contrast to suberoylanilide hydroxamic acid (SAHA)-inducible apoptotic cell death in the cell line MES-SA, ESS-1 was found to undergo autophagy-associated cell death due to a homozygous nonsense mutation in the *TP53* gene that causes p53 protein deficiency or degradation [49,50].

Autophagy is furthermore activated in response to an increasing number of drugs used in cancer treatment to protect against cellular stress. This protective function of autophagy can be considered as a mutual response of the cell that prevents both, induction of either apoptotic or necrotic cell death [36,51,52]. As a quite often realized problem, however, autophagy also facilitates resistance of the tumor cell to chemotherapy and radiation treatment [46,53]. To avoid and re-sensitize therapeutic resistant cancer cells, several strategies deploy the use of inhibitors as an adjuvant therapy, such as 3-methyladenine (3-MA), the lysosomotropic substance chloroquine (CQ) or its analog, hydroxychloroquine (HCQ), as well as BafA1 (bafilomycin A1), or use gene silencing that abrogate autophagy [54,55,56,57]. In fact, drug mediated inhibition of autophagy in combination with cytotoxic drugs constitutes a novel potential target in cancer therapy [31,47,58,59,60,61,62]. Nevertheless, in the case of histone deacetylase inhibitor (HDACi) treatment which is intended to promote alternative modes of cell death this can result in a drawback; tevidently, in the presence of apoptosis resistance, disruption of autophagy, will evoke enhanced tumor survival as the only escape route is blocked. This should again underline the necessity of affirming the context-dependent role of autophagy in cancer treatment by previous investigation of the limiting molecular determinants, such as oncosuppressors and oncoproteins [37,47,63,64,65]. 

## 3. Autophagy as a Therapeutic Target in Drug Resistance

Increasing evidence implicates an important role of autophagy in developing resistance to chemotherapeutic treatment of cancers [37,66,67,68,69,70]. This is a particular drawback in the therapy of tumors that are anyway hard to treat. A multiple spectrum of different cancer drugs has been demonstrated to activate autophagy which in several cases by eliciting therapeutic resistance also facilitates survival of tumor cells [31,63]. Thus, interrelated autophagy induction and drug resistance have been observed in ovarian cancer treated with paclitaxel, in ovarian and esophageal cancer teated with cisplatin, in lung cancer following hypoxia, in melanoma treated with cyclin dependent kinase (CDK) inhibitors, or in glioblatoma cells treated with the HDACiTSA, to just name a few (reviewed in [31]). 

Acquired resistance will be indicated by increased levels of autophagy that was indeed observed in several cases, and therefore can be tackled by the additional use of autophagy inhibitors such as CQ. In addition to pharmacological inhibitors, re-sensitization of autophagy can also be regained by genetic inhibitors, which could target several different concerned pathways of acquired autophagic resistance as documented in vitro and by pre-clinical studies [63]. This strategy can either target specific pathways of autophagy or block the entire process to obtain a clinical benefit. Specifically, due to the frequent link between drug-mediated induction of autophagy and chemotherapeutic resistance, the suppression of autophagy presents in return most evidently a promising target in cancer therapy. For example, in estrogen receptor-positive breast cancer, inhibition of autophagy sensitized resistant tumors to tamoxifen-induced killing [71,72]. Similarly, in prostate cancer, autophagy inhibition overcame resistance to enzalutamide [73]. Autophagy is also induced in response to treatment of gastrointestinal stromal tumor (GIST) cells with Imatinib™ and inhibition of autophagy (by CQ) caused tumor cell apoptosis [74]. 

As one of the most obvious molecular explanations for the chemotherapy-associated activation of autophagy, decreased mTOR protein levels have been held responsible which could result from therapies that target either mTOR directly or via the interrelated pathway activities of PI3K or AKT [75]. De-repression of autophagy during genotoxic treatment of cancers however, which include cisplatin or radiation treatment, might be attributed to the DNA-damage-regulated actions of p53. In this sense, the nuclear stress-activated transcription factor p53 can directly mediate autophagy (and apoptosis) via the damage-regulated autophagy modulator (DRAM) which represents a lysosomal protein that is able to advance different stages of autophagosome formation [76,77]. Recently, even the activity of microRNAs has been implicated in modulating drug resistance via regulation of ATG genes which underlines their crucial role in autophagy [78]. Nevertheless, evidence for molecular details of the direct autophagy-causing activities of other cancer therapeutics is lacking thus far. As an alternative explanation it has been suggested that by various treatment therapies, cancer cells could be specifically selected that already undergo high levels of autophagy which were named therapy refractory cancer stem cells. Thus, in different types of tumors following irradiation, higher levels of autophagy were detected which responded to inhibition of autophagy by reduced survival of breast, lung and cervical cancer cell lines [55].

## 4. Histone Deacetylase Inhibitors and Their Effector Mechanisms

Deregulated histone and non-histone deacetylation as a major epigenetic regulatory mechanism of gene transcription has been recognized as the underlying cause of transcriptional deregulation of tumor-related genes in many solid as well as hematological tumors [79,80,81]. By impeding crucial physiological cellular processes related to cell death regulation, cell proliferation, differentiation, senescence, migration and adhesion, malignant transformation of the cell is facilitated [82,83,84,85,86]. In order to support the cancer cell in resisting imbalanced growth-promoting and cell death signals, in recent years HDACi have been promoted as encouraging anticancer agents to interfere with epigenetically deregulated gene. 

**Figure 1:**
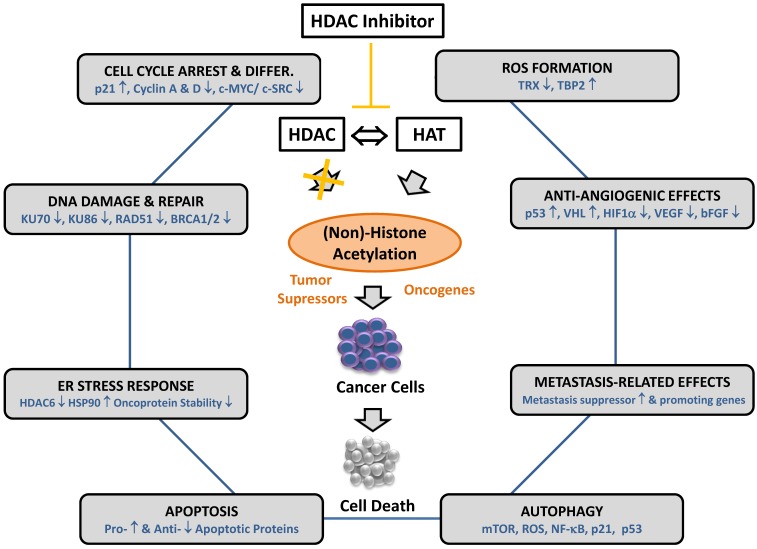
Overview about major effector mechanisms of histone deacetylase (HDAC) inhibitor (HDACi) in cancer cells. Important individually affected molecules (blue font) of the different categories of HDACi- activated anti-tumor pathways (grey boxes) are displayed. HDACi interfere with the deacetylation of histone and non-histone protein deacetylation of many tumor tuppressor genes and oncogenes (among other regulatory proteins) in cancer cells; this posttranslational modification renders them active and thereby induces the different effector mechanisms eventually causing cell death. ↑ upregulation or activation; ↓ downregulation or inhibition; HAT, histone acetyl transferase(s); ROS, rewactive oxygen species; TRX, thioredoxin; TBP2, TRX-binding protein 2; p53, tumor suppressor protein p53; VHL, von Hippel Lindau factor; HIF1α, hypoxia-inducible factor 1α; VEGF, vascular endothelial growth factor; bFGF, basic fibroblast growth factor; mTOR, mammalian target of rapamycin; NF-κB, nuclear factor lappa B; p21, cyclin-dependent kinase inhibitor 1 (p21^CIP/WAF1^); HDAC6, histon deacetylase 6, *c-MYC*, cellular homolog of the oncogene (*v-myc*) of avian myelocytomatosis virus strain 29; *c-SRC*, cellular SRC kinase oncogene; ER, endoplasmatic reticulum; Ku70, Lupus Ku autoantigen protein p70; Ku 86, Lupus Ku autoantigen protein p86; RAD51 (BRCC5); DNA repair protein RAD51 homolog; BRCA1/2, BRCA1/2, DNA repair associated (prev. breast cancer suceptibility protein ½).

Expression caused by aberrant post-translational protein modifications. Similar to well-established epigenetic drugs, as for example DNA methyltransferase inhibitors, HDACi reverse histone acetyltransferase- and deacetylase-mediated acetylation of both, histone and non-histone proteins. Although HDACi-mediated cytotoxic responses are very pleiotropic, and seemingly depend on the type of tumor and used drug doses, specific re-occurring mechanisms are common for many HDACi that ultimately trigger tumor cell death [87,88,89] (Figure 1). Major effector mechanisms of HDACi are found in the ability to re-induce cell cycle arrest by p53-dependent or -independent induction of the cyclin-dependent kinase inhibitor p21^CIP/WAF1^ (p21). These effects induce cell differentiation in transformed cells by downregulating positive regulators of cell proliferation, such as *c-MYC* and *c-SRC* [90,91,92,93,94,95,96,97]. It is assumed that accumulated DNA damage in arrested tumor cells then promotes the activation of apoptosis or mitotic cell death as it cannot exit the cell cycle and finish mitosis [98,99,100,101]. This mechanism is furthermore underscored by the ability of HDACi to enhance the formation of ROS possibly due to the exclusive suppression of the natural scavenger thioredoxin (TRX)- an intracellular antioxidant- via upregulation of TRX-binding protein 2 (TBP2) in malignant cells [102,103,104]. Other HDACi anticancer mechanisms cause interference with stress response pathways of the endoplasmic reticulum, affect chaperone protein function leading to altered stability and expression of oncoproteins, or facilitate the accumulation of misfolded proteins [105]. In addition, HDACi-mediated de-repression of metastasis-related genes, influencing migration and invasion behavior of human tumor cell lines, as well as altered angiogenic inhibition, due to re-expressed pro- and anti-angiogenic genes, was documented [106,107,108,109,110,111,112,113,114,115]. Moreover, histone deacetylases (HDAC) have been involved in directing the expression of DNA damage-related response proteins and the modulation of histone deacetylation at sites of DNA damage to maintain genomic integrity; thus, only recently, HDACi administration has been found to interfere with a range of DNA repair processes in response to the induction of autophagy by genomic damage [79,116,117,118,119].

Key determinants of HDACi-triggered lethality are apoptosis and autophagy in a multitude of tumor cells; due to re-expression of silenced tumor suppressor genes and/or oncogenes, inhibitory signals for cellular growth and elevated cellular stress are (re-)activated. Accordingly, direct modulation of the acetylation pattern of transcription factors belonging to the class of non-histone proteins, can re-activate cell death-related signaling pathways in transformed cells (e.g., NF-κB, p53, and STATs) [85]. For example, HDACi treatment determines the acetylation status of the transcription factor p53 and thereby influences its mutual direct interaction with the MDM2 E3-ligase which regulates its degradation in H1299 carcinoma cells [120].

## 5. Resistance to HDAC Inhibitor-Induced Autophagy

**Figure 2:**
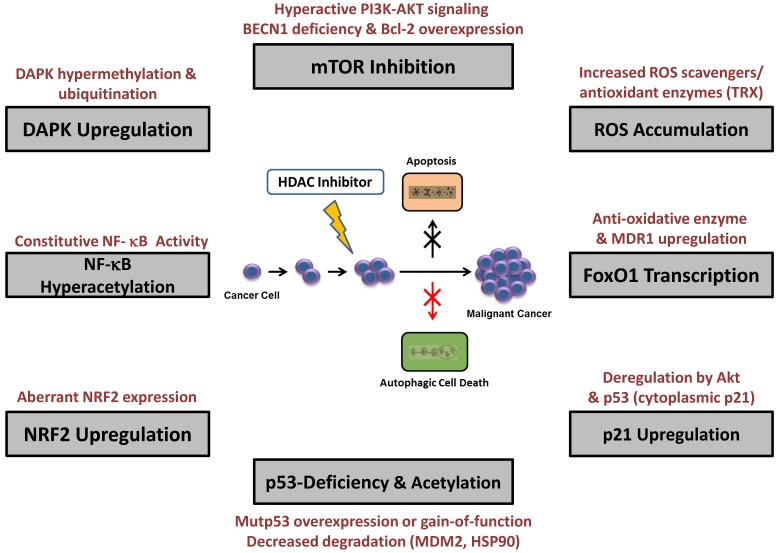
Different mechanisms of histone deacetylase (HDAC) inhibitor-induced autophagy (grey boxes) and presumed molecular determinants that cause resistance (red font). As many cancer cells already block many pathways leading to apoptosis (black arrow), which cannot be reversed by HDACi treatment, the additional occurrence of resistance to autophagy or the additional use of autophagy inhibitors (red arrow) eventually facilitates cancer cell survival. BECN1, BECLIN-1; Mutp53, mutant p53; MDM2, mouse double minute 2; HSP90, heat shock protein 90; TRX, thioredoxin; MDR1, multiple drug resistance-1.

Treatment with HDACi has exposed several varying molecular signaling mechanisms leading to the activation or suppression of HDACi-mediated autophagy (reviewed previously in [121,122,123,124,125,126,127] Figure 2 and Table 1). In by far most cases, mTOR inhibition involving the canonical pathway, followed by ROS accumulation, NF-κB hyperacetylation, p21 upregulation, or the involvement of p53 signaling, is observed during autophagic activation [49,58,128,129,130,131,132,133,134,135,136,137,138,139,140,141,142]. Individual reports of HDACi-induced autophagy also detail apoptosome inactivation, increased transcriptional activity of FoxO1 (forkhead box O1) or NRF2 (Nuclear factor erythroid 2-related factor 2), upregulation of DAPK (death-associated protein kinase) and nuclear translocation of AIF (apoptosis inducing factor) [143,144,145,146,147]. Nevertheless, beside these defined mechanisms, direct HDAC-mediated acetylation of many ATG proteins, including ATG3, ATG7 or ULK1 (UNC-51-like kinase 1) has been brought in evidence, in addition, or as the only findings [85,148,149,150]. HDACi-mediated suppression of autophagy additionally involved mTOR attenuation in two reports [64,151,152].

HDACi-elicited autophagy has also been linked with the occurrence of therapeutic resistance [58,185,186,187,188]. Consequently, a discussion is ongoing, whether induction of autophagy stimulates antitumor activity or assists in the development of chemotherapeutic or radiotherapeutic resistance; the later might be true as malignant cells are exceedingly adaptable to inhibitory survival stimuli and cytotoxic agents [189,190]. As a result of permanent selection during tumor development and chemotherapy, particularly cancer cells are finally able to deal with a range of harmful conditions such as oxidative stress or DNA damage that renders them independent from external growth signals. In addition, stable genetic or epigenetic alterations of tumor cells that initially trigger tumorigenesis and particularly modify death signaling, might also be detrimental factors that inevitably determine the outcome of the autophagic response. Considering the increasing number of clinical trials performed the identification of presumable counteractions of potential drug combinations and the improvement of their efficacy gains significant weight. 

This is particularly necessary, as only few in vivo data are available and research focused on only a few multidrug-resistant cell lines (linked to P-glycoprotein) [191,192,193]. Previous studies underscored molecular mechanisms, such as alterations in HDAC protein expression, retinoic acid signaling deregulation, and multidrug resistance facilitated by ATP-binding cassette transporters (*MDR1* and its product, P-glycoprotein) as emanating cause of HDACi resistance [186,194,195,196]. As a predictive biomarker, constitutive STAT (signal transducers and activators of transcription) activation has been reported in SAHA (vorinostat)-treated CTCL patients [197]. Related to the identified triggering signals of HDACi-elicited autophagy, we summarize below the characterized and potential molecular basis of resistance mechanisms (Figure 2 and Table 1).

### 5.1. mTOR Modulation

The nutrient-sensing kinase mTOR (mammalian target of rapamycin) is a primary suppressive coordinator and upstream key component of the autophagic signaling machinery that regulates the ULK1 complex [3,4,198]. mTOR associates with unique binding partners and thereby reacts and controls, as a central interface of growth factor and nutrient signaling, many cellular functions via two multiprotein complexes, mTORC1 and mTORC2; this involves, for instance, proliferation, growth, survival, motility, and metabolism [199,200,201,202,203]. Due to the versatile modulation of the mTOR complexes, the stimulation and suppression of autophagy can be achieved in many ways. Thus, they are controlled on the one hand by PI3K (phosphatidylinositol 3-kinase)/AKT (a serine-threonine kinase encoded by the human homolog of the oncogene in the transforming retrovirus, AKT8; also called protein kinase B (PKB) but also by AMPK (AMP-activated kinase). AMPK acts as a central sensor for energy and metabolism (such as lipid, cholesterol and glucose metabolism) that is specifically controlled by Rag GTPases for amino acid availability, by REDD1 for oxygen availability, and by p53 for DNA damage in the cell [204,205,206,207]. During times of sufficient supplies, initiated by the phosphorylation of AKT, mTORC1 generally suppresses the activation of autophagy by phosphorylating itself the ULK1 complex further downstream, and only basal autophagy can take place [208,209]. During conditions of nutrient deprivation or shortage, the induction of autophagy is essential for cell survival, as cellular components can be reused and provide metabolic supply. In this event, AMPK can be activated by phosphorylation mediated by its potential main upstream regulator, the serine-threonine kinase LKB1 (liver kinase B1) that supports activation of the tumor suppressor tuberous sclerosis protein (TSC2) [210]. GSK3 (Glycogen synthase kinase-3)-initiated activation of the histone acetyltransferase TIP60 and consequent acetylation of ULK-1 represents a further possible mechanism of starvation-induced autophagy [211]. mTORC1, that can be inhibited by rapamycin, plays also a detrimental role in regulating protein translation by controlling the phosphorylation of the elongation factor 4E-BP1 and p70S6 kinase [212]. These two factors are presumed to mediate mTOR-induced suppression of autophagy [213,214].

As a further major downstream player in the canonical mTOR-activated autophagic signaling cascade-mediated by ULK1, Beclin-1 is required for the organization of autophagosome formation [160]. Beclin-1 enables autophagy mainly through forming extensive protein complexes with other autophagy-related proteins belonging to the Beclin-1 interactome (e.g., Vps34, p150, UVRAG, Bif1, Atg14L, Rubicon) [215]. Modifications of transcriptional regulation of pro-apoptotic (BAX, BAD, BNIP3, or PUMA), or anti-apoptotic members (BCL-2, BCL-XL, and MCL-1) of the BH3-only family, inhibit or activate Beclin-1-dependent autophagy by direct interaction with the BH3 domain of Beclin-1 [216,217,218,219]. 

The PI3K/AKT/mTOR signaling axis presents comparably one of the most frequented pathways of HDACi-activated autophagy that is often supported by increased ATG protein expression, such as LC3 (microtubule-associated protein 1A/1B-light chain 3) and Beclin-1 [49,135,136,137,138,139,151,153,154]. SAHA-mediated inactivation of mTOR, for example, induces autophagy by restoring the function of the ULK1 complex [134,136,137,138,139]. Initially, this undispensable role of mTOR in the regulation of SAHA-induced autophagy was experimentally verified by studies of our own group on uterine sarcoma cells and by Gammoh et al. [49,136,153]. Nevertheless, the ULK1 complex has an adequate function in mediating HDACi signaling, as ULK1-deficient cells have been documented to block SAHA-elicited autophagy [136]. Interestingly, two cases also reported about HDACi-elicited suppression of autophagy by activating the PI3K/AKT/mTOR pathway. In the first case, MGCD0103 enhanced the degradation of autophagy-related proteins in primary CLL (chronic leukocytic leukemia) cells via silencing *ATG7* expression [151,152]. In the second report, accumulation of mitochondria and ROS, DNA damage, and apoptosis were detected in down syndrome-associated myeloid leukemia cells (DS-AMKL cells) that were treated with various HDACi (SAHA, TSA (trichostatin A), VPA (valproic acid), MS-275 (etinostat), JQ2) resulting in suppression of baseline autophagy due to activation of mTOR [64]. 

Considering the emanent role of the PI3K/Akt/mTOR signaling cascade in controlling essential cellular functions, it is not an unanticipated finding, that in many cancers dysregulation can be found in this pathway [220,221]. Thus, therapeutic resistance can be caused for instance by constitutive PI3K-AKT signaling that hyperactivates mTOR and therefore inhibits autophagy, which is encountered in many cancer cells; this is often due to mutations in PI3K itsself or other upstream signaling factors, loss of the tumor suppressor genes PTEN (phosphatase and tensin homolog) and ARHI, or by AKT overexpression [155,156,157,158]. Particularly, about 60% of breast cancer tumors accommodate genetic variations leading to high activity of the PI3K/AKT/mTOR signaling route which have been classified as oncogenic driver mutations [222]. Consequently, many targeted therapeutic interventions that address the reversal of endocrine resistance, apply inhibitors for mTOR (such as rapamycin (sirolimus) or its rapalogs temsirolimus, everolimus, deforolimus, etc.), for PI3K (buparlisib, pictilisib, copanlisib, alpelisib, and taselisib), and for AKT (ipatasertib) which entail diminished mTOR activity and therefore derepression of autophagy [223]. Due to off-target inhibition and adaptive autophagic resistance, it is not surprising that major challenges in developing optimized and rational drug combinations for the PI3K/AKT/mTOR signaling pathway persist.

Resistance to Beclin-1-mediated autophagy arises mainly due to monoallelic loss of the tumor suppressor protein Beclin-1, particularly in sporadic breast, ovary, and prostate tumors, or due to overexpression of the oncoprotein Bcl-2 related to an aggressive clinical tumor phenotype in a variety of cancers [160,161,162,163]. Thus, direct obstruction of Bcl-2 mediated autophagic cell death was documented in breast cancer cells which could be overcome by gene silencing, thereby offering a novel therapeutic strategy [218,224].

### 5.2. ROS Accumulation and Enhanced Antioxidant Expression 

ROS are induced by various kinds of cellular stress and disturb the mitochondrial respiratory chain and energy metabolism. Due to this activity, they have frequently also been involved in ROS-induced dual activation of apoptosis and autophagy [225]. Here, selective autophagy in the form of mitophagy-regulated by NIX/BNIP3L- and PINK1-mediated pathways represents a tumor suppressor mechanism, as the elimination of damaged mitochondria prevents the further accumulation of ROS [226,227,228]. Mitochondrial damage and endoplasmatic reticulum stress resulting in the formation of ROS might furthermore lead to an autophagic response via activation of transcription factors such as FOXO and ATF4 that in turn increase the expression of ATG genes (*LC3*, *ATG5*) [229,230,231]. A further mechanism of ROS-induced autophagy could be mediated by Ataxia-telangiectasia mutated (ATM), a cellular damage sensor that coordinates the cell cycle, with damage-response checkpoints and DNA repair, to preserve genomic integrity [232]. In response to elevated ROS, ATM activates TSC2, via the LKB1/AMPK metabolic pathway in the cytoplasm, to repress mTORC1 and induce autophagy.

ROS induction represents a further common factor that initiates the activation of HDACi-mediated autophagy in tumor cells [233]. It has been reported to occur as a single event or in combination with mTOR deactivation, due to extensive intracellular ROS generation [122,123]. Upon deleting *ATG5* or *ATG7* in cells, the use of autophagy inhibitors in the presence of ROS has been demonstrated to promote tumor development, due to the culmination of deleterious mitochondria and chronic oxidative stress as well as tissue damage and inflammation [234,235,236]. 

The specific mechanism leading to HDACi-induced accumulation of ROS has not been resolved up to date but seem to lie in the posttranslational modification of TRX, representing an intracellular antioxidant that serves as a natural scavenger of ROS. Corresponding evidence was derived from the finding that increased TBP-2 expression could be localized in normal but not in malignant fibroblasts in humans which decreases the reductive capacity of TRX [102]. Thus, tumor cells treated with HDACi have a lowered ability to handle oxidative injury. This assumption is consistent with the analysis of several tumors where, in addition to ROS generation, activation of the MAPK (mitogen activated protein kinase) family members, ERK1/2 and JNK or elevated expression of the lysosomal protease cathepsin D in combination with diminished levels of its substrate, TRX could be detected [58,137]. In the case of MS-275 treated human colon cancer cells, p38 MAPK even played a vital role in switching between HDACi-mediated apoptosis and autophagy [166,237]. In one report however, HDACi (VPA, TSA)-mediated autophagy and cytotoxicty occurred in pancreatic cancer cells obviously due to concomitant increased ROS production and HDACi-induced ERK1/2 inhibition which promoted *c-MYC* downregulation, a survival factor for cancer cells exhibiting constitutively activated RAS [130]. This depicts the upregulation of enzymes related to cellular redox control, anti-oxidative stress, and energy metabolism in the proteomic study of SAHA-induced Jurkat T-leukemia cells [137]. Contrastingly, ROS-mediated autophagy promoted survival of AML1-ETO cells in response to SAHA and VPA treatment due to the removal of ubiquitinated proteins; this could be overcome by the use of HDACi and CQ cotreatment; dependency on ROS for the mediation of cell death could be clearly demonstrated by the use of the ROS scavenger N-acetylcysteine (NAC) in these cells [62].

Several anticancer therapies that stimulate ROS- induced autophagy and possibly apoptosis have also been linked with the development of cell death resistance [44,59]. As many cancer targeting drugs also promote cellular stress, the increased formation of ROS could also upregulate tumor cell-protective levels of autophagy that facilitate cancer cell survival. In the case of ROS-induced autophagic resistance, one could therefore also postulate that some classes of HDACi might not exert a perfect suppressive effect on TRX expression levels; this might be particularly relevant for cancer cells where apoptosis is not activated in parallel by the used HDACi. Accordingly, increased levels of ROS scavenging and anti-oxidant enzymes have been detected in multidrug-resistant cancer cells [164]. In this case, it is hard to predict whether the use of autophagy inhibitors might restore the sensitivity to the treatment thereby modulating cancer cell proliferation, survival, and drug resistance.

### 5.3. p21^CIP/WAF-1^ Upregulation 

Many HDACi exert antiproliferative effects by re-inducing arrest in the G1 or G2 phase of the cell cyle and by inducing differentiation through upregulating p21 expression [93,238]. The consequent cyclin-mediated inability to pass two cell-cycle checkpoints is presumed to exert a protective function on the transformed cell allowing its repair. In the case of accumulated DNA damage in tumor cells (such as DNA double-strand breaks), the inability to further proceed in the cell cycle causes an early escape from an unfinished mitosis and the subsequent induction of cell death [95,98,100,101,239]. *CDKN1A*, the gene enoding for p21, is one of the most prominent target genes of p53, nevertheless, also p53-independet induction of p21 has been reported. Acetylation and HDAC1-mediated deacetylation of p53 have been shown to mutually regulate binding of p53 to the promoter region of *CDKN1A*, and its expression [238,240]. p53 is also the main regulator of p53R2, a human ribonucleotide reductase homologue that contains a p53-binding site in intron 1 which up-regulates p21 [241].

HDACi-mediated upregulation of p21 can promote cell death via apoptosis, and in two reported cases also via autophagy, but the underlying mechanism is poorly resolved. In the first case, p21-mediated autophagy occurred in addition to cell differentiation and cell cycle arrest; this was mediated by the HDACi combination of H40 and SAHA resulting in hyperacetylation of histone H3 and upregulated p21 expression in PC-3M and HL-60 cells [142]. In the second event, the HDACi MRJF4 was found to induce p21-elicited autophagy in PC3 cells [128]. In both studies, additional downregulation of NF-κB signaling was also documented, which could be assumed as the primary cause of autophagic induction. In a further study of apicidin-induced autophagy in human oral squameous cell carcinoma cells, p21-mediated G2/M phase arrest was accompanied by mTOR attenuation [134,159]. In two reports of uterine and pancreatic cancer cell autophagy, concomitant with either p53-deficiency or HDACi-induced mutant p53 induction, upregulation of its targets p21 could be observed [49,130].

Cytoplasmic localization of p21, possibly due to deregulated Akt1-mediated phosphorylation or truncation of the nuclear localization signals has been related with an anti-apoptotic gain-of-function [172,173]. The anti-apoptotic function of p21 includes inhibition of caspases, such as CASP3, 8, and 10, stress-activated protein kinases (SAPKs), and apoptosis signal-regulating kinase 1 but can also involve the up-regulation of anti-apoptotic protein encoding genes [172,242]. As cytoplasmic p21 has been frequently identified in aggressive tumors that are particularly associated with poor prognosis, this finding could nevertheless also represent a cause of autophagic resistance [174]. Deregulation of Akt can thereby occur through genetic modifications of PI3K, but also by loss of PTEN, or by HER2/neu (ERBB2) amplification. Moreover, p53-mediated upregulation of p21 via p53R2, also targets cytoplasmic p21 and thereby facilitates the progression of cancer [241]. Consistently, p53 and p53R2 may have a tumor-promoting function in cancers with cytoplasmic p21 (and Akt overexpressing cancers), and support the development of chemoresistance against anticancer therapeutics leading to poor prognosis. Furthermore, radioresistance via DNA damaging therapies, such as UV irradiation, can be obtained in this way as the p53R2-p21 interaction is weakened and levels of non-bound nuclear p21 increases [175]. Consequently, also various forms of mutated or deficient p53 and its regulatory proteins are able to cause p53R2-mediated deregulation of p21 and the formation of resistance against HDACi-induced cell death.

### 5.4. p53 Deficiency and Acetylation

The initial identification of p53 as an important regulator of autophagy was established by the clarification of its target gene DRAM1 (damage-regulated autophagy modulator). DRAM not only directly activates apoptosis, but also autophagy, when DNA-damaging agents induce p53 transactivation [76]. It represents a lysosomal protein that is entangled in different phases of autophagosome formation and could also be activated by the p53 relative, p73 [77,243]. Positive transactivation-dependent regulation of autophagy involves furthermore p53-mediated activation of the tumor suppressor genes genes TSC2 (tuberous sclerosis complex 2) and PTEN (phosphatase and tensin homolog), as well as AMPK, or its activators sestrins 1 and 2 [157,244,245,246]. The further signaling cascade of these pro-autophagic factors then follows the canonical pathway of autophagy, mediated by mTOR attenuation. p53 can furthermore influence the expression of pro-apoptotic proteins (downregulation of BCL-2, BCL-xL, and MCL-1), anti-apoptotic proteins (upregulating BAX, BAD, BNIP3, or PUMA), or the tumor suppressor protein p14ARF (alternate reading frame protein product of the CDKN2A locus) leading to the release of Beclin-1 [218,247,248]. p14ARF seems to be important in stabilizing the p53 protein and maintaining its cell death regulation ability, in order to oppose hyperproliferative signals causing oncogenic activation [249,250]. As a further p53-regulated protein, DAPK induces autophagy, either by phosphorylating and stabilizing Beclin-1 against BCL-2/BCL-xL-mediated degradation, or by blocking the anti-autophagic activity of the LC3-interacting MAP1B protein [217,251,252]. Negative regulation via p53 protein, located in the cytoplasm, has been additionally uncovered at last [253,254,255,256,257]. Although the exact mechanism still needs to be elucidated, this inhibitory function is a transcription-independent process that finally also targets the identical AMPK-mTOR signaling cascade used by nuclear p53 transactivation-dependent autophagy. Under normal physiological conditions, in contrast to nuclear p53 activity, direct interaction of p53 protein with AMPK increases mTOR activity, leading to autophagic suppression [256]. This tumor-suppressive mechanism, resulting in increased autophagic flux in p53-deficient cells, is presumably intended to provide elevated resistance to metabolic stress. mTOR-independent inhibition of autophagy by cytoplasmic p53 has been furthermore shown to be mediated by TIGAR (TP53-induced glycolysis and apoptosis regulator) resulting in downmodulation of glycolysis and of ROS formation, as a response to stress [258].

HDACi-dependent autophagic induction involving p53 was previously reported by our own study, that revealed p53 deficiency as the cause of mTOR signaling-mediated autophagy in SAHA-treated ESS-1 uterine sarcoma cells, and in MDA-MB-231 breast cancer cells by Fogetti et al. [49,122,123,237,259]. Similarly, to our finding, the HDACi VPA and TSA provoked, in response to p53 deficiency, concomitant upregulation of apoptosis and autophagy in pancreatic cancer cells [130]. However, opposed to homozygous inactivation of *TP53* in ESS-1, cell death activation was mediated by reduced mutant p53 expression in this case due to ERK-mediated stabilization of the oncogenic protein c-MYC, and by reactivation of wild-type p53 expression. Increased acetylation of the p53 protein following specific inhibition of class III NAD-dependent deacetylases SIRT1 and SIRT2 by sirtinol or combined sirtinol/MHY2256 treatment, preventing MDM2-mediated degradation, yielded elevated LC3-II expression and autophagy, in addition to cell cycle arrest and apoptosis in MCF-7 breast cancer cells or in endometrial cancer cells, respectively [131,132,133].

While intact p53 protein is essential for maintaining stress-mediated responses and DNA repair in the cell, impairment of its integrity by mutation is frequently found in almost all types of human cancers [260]. Altered p53 functions, most commonly due to missense mutations, favor malignancy and resistance to chemotherapy. These genetic alterations lead to a loss in the trans-activation capability of the transcription factor rendering the cell vulnerable against applied stress and malignant transformation; alternatively, they generate dependence on alternative pathways or proteins required for cell survival. Depleting these redundant survival pathways in p53-deficient cells is therefore one of the objectives of future cancer treatment. Overly resistance to chemotherapeutic drugs, including cisplatin, alkylating agents, anthracyclines, antimetabolites etc., was often related to overexpression of mutant p53 that possesses conformational alterations. Thus, mutant p53-reactivating small molecule compounds such as CP-31398, WR-1065, PRIMA-1, and MIRA-1, or the blockage of proteasomal degradation might help to restore p53-dependent cell death and tumor suppression [261,262,263,264,265,266]. Frequently, however, these heterozygous mutations acquire, due to hyperstabilization and accumulation, dominant-negative or gain-of-function activity over p53 protein, derived from the intact allele (e.g., by impeding access to the promoter), which finally confers pro-ongogenic roles; these range from malignant cell proliferation and invasion to the development of drug resistance [167,168,169]. Additionally, or due to these gain-of-function mutations, p53 activity may be also be defective caused by alterations in regulatory proteins of p53, particularly of MDM2 that determines its half life. Reduced mutant p53 degradation is therefore facilitated by overexpressed short isoforms of MDM2 or auxillary chaperone proteins (such as HSP90, BAG family proteins) [170,171]. 

As one of the effector mechanisms, it was elucidated that the interaction of HDACi with mutant p53 protein enables upregulation of p21 and MDM2, and thus its proteasomal degradation [267,268,269]. This could be demonstrated by treating heterozygous p53-deficient cells with TSA, FR901228, or SAHA, which re-established p53-dependent transcription, either by re-activating wild-type expression of p53 itself, by copying p53 transcriptional activity, or by upregulating autophagy [130,268,270]. In a further report, release of mutant p53, by SAHA-facilitated disruption of a complex consisting of HDAC6 and heat shock protein 90 (HSP90), enabled MDM2 and CHIP ligase-mediated degradation [269]. Additionally, the transcription factor HOXA5 was documented to upregulate mutant p53 mRNA and protein expression in tumor cells following SAHA, NaB (sodium butyrate) or HDAC8 administration [271]. Very recently, HDACi-mediated degradation of mutant p53, through the activation of autophagy, has also been reported. In the human breast cancer lines MDA-MB-231 (mutp53-R280K) SAHA induced significant depletion of mutant p53 supporting tumor survival that could be blocked by inhibition of autophagy leading to increased cell death [259]. Also in our previously described model of the uterine sarcoma cell line ESS-1, the activation of SAHA-induced autophagy could be explained by elimination of mutant p53 (mutp53-R213X) and the lack of p53 protein; although not yet experimentally verified, which could target the cytoplasmic p53-mediated negative regulatory pathway of autophagy [49]. These findings support the anticancer strategy of engaging cellular pathways that facilitate mutant p53 degradation which may not only reduce the oncogenic potential of cell growth and invasion but also increase the sensitivity to anticancer drugs. Accordingly, beside HDACi, DA several encouraging HSP90 inhibitors (such as 17-AAG or ganetespid) and MDM2 inhibitors (nutlins, benzodiazepinediones or spiro-oxindoles) have been proposed that affect the p53-MDM2 signaling axis, and therefore open new possibilities for the development of more efficient anticancer drugs [272,273]. 

### 5.5. NF-κB Hyperacetylation

In addition to their well-known role in regulating immune responses, the NF-κB (Nuclear factor kappa B) transcription factor members (RelA, RelB, c-Rel, NF-*κ*B1, and NF-*κ*B2) are critical anti-apoptotic factors that sustain cell growth and survival and are frequrently deregulated in cancer. Under normal conditions, proteins of the NF-κB family are kept inactive by several inhibitory cytoplasmic proteins belonging to the I*κ*B kinase complex [274,275]. In tumor cell lines, NF-κB has been furthermore established as a suppressive regulator of autophagy, in response to the cytokine TNFα, which may contribute to suppression of TNFα-induced apoptotic signaling and oxidative stress. Following the induction of NF-κB mediated autophagy, the autophagy-associated proteins Beclin-1 and p62/SQSTM1 were found upregulated; IKK kinase in contrast, an activator of NF-κB was degraded by autophagy, due to a loss of Hsp90 function, leading to the inhibition of NF-κB signaling. The exact mechanisms of autophagic induction are unknown but could involve mTOR signaling or interference with Beclin-1/Bcl-2 stabilization. Conversely, inhibition of NF-κB signaling was reported to augment starvation-induced autophagy [276]. Interestingly, TSC2, an inhibitor of the mTOR pathway, positively regulated NFkB activity revealing a reciprocal relationship between NF-κB and autophagy that might represent a feedback loop of cell death signaling. 

NF-κB-mediated autophagy induced by HDACi has been reported in three studies. Combined SAHA and MS-275 treatment of PC3 cells positively regulated autophagy by by NF-κB RELA/p65 hyperacetylation leading to transcription of several NF-κB target genes and suppressed the innate immune system in vesicular stomatitis virus oncolysis [141]. In two reports, induction of autophagy was preceded by suppression of pERK/NF-κB signaling and upregulation of p21 in PC-3M (and HL-60) cells [128,142].

NF-κB and its activating kinase, IKK, have attracted therapeutic intervention because of their significant function in the progression, not only of cancer, but also of many other diseases involving chronic inflammation. Besides the regulation of cell death, NF-κB signaling has critical immunomodulatory roles in arranging cytokine release, regulating prostaglandins and redox reactions, and in governing angiogenesis [277]. NF-κB upregulation or constitutive active signaling, in cancer or normal cells, suppresses apoptosis, wheras it promotes angiogenesis and metastasis thereby causing resistance to radiotherapy and chemotherapy which reduces therapeutic efficiency. Nevertheless, as many other pathways convene at the NF-*κ*B pathway, including those of other immune-chemotherapeutic triggered phenotypes of resistance, it is hard to determine the specific molecular culprit [176]. Either the application of selective inhibitors, or the use of natural NF-κB agents (such as curcumin, melatonin, resveratrol, and quercetin), have shown encouraging results for tumor sensitization but need to be verified in clinical studies.

### 5.6. FOXO1 Transcription 

FOXO1 is a member of the FOXO family that serve as transcriptional regulators in many intracellular processes, such as cell growth, proliferation, differentiation, and longevity, including regulation of autophagy [278]. Via post-translational modification of FOXO1, the modulation of distinct sets of target genes can be activated that execute functions in silencing oxidative stress by regulating anti-oxidative enzymes, metabolic and immune effectors, DNA repair, cell cycle arrest and apoptosis, as well as autophagy-associated genes [279,280,281]. Involved triggers of FOXO1 activity include oxidative stress, high glucose, and several more, that are mediated by PI3K-AKT, JNK, CBP, sirtuins (SIRT), or ubiquitin E3 ligases signalling pathways [282]. FOXO1-driven autophagy or apoptosis has been demonstrated as an important function in response to stress; it is regulated by acetylation of FOXO1 following its dissociation from SIRT2—a mainly cytoplasm-located deacetylase- and by subsequent binding to ATG7, a key regulator in the formation of the autophagosome [245]. But acetylation of FOXO1 and activation of autophagy can also be triggered by the natural agents benzyl isothiocyanate and curcumin, as well as by starvation [283,284]. 

FOXO1-stimulated transcriptional activation of SAHA and TSA-treated HepG2 and HCT116 cells was also declared as a mediator of HDACi-induced autophagy [145]. As underlying mechanism, sestrin 3 (SESN3)-mediated suppression of mTOR and TSC2, in addition to upregulation of ATG gene expression (such as *ATG4B*, **ATG**12, *PIK3C3*, *BECLIN-1*, and *MAP1LC3B*), could be detected. Due to their commonly altered expression in many tumors, cytosolic FoxO1-modulated cell death is considered as tumor suppressor activity. Regulatory pathways involved in the oncogenic activity of FOXO factors have been including PI3K, Ras, IKK signaling but also microRNAs and have been linked to the deregulation of cell cycle arrest (p27_KIP1_, p21) and cell death genes (e.g., *FASL*, *TRAIL*, *BIM*) [285,286]. 

Nevertheless, recent reports elucidate a more intricated role of FOXO factors by contributing to cancer proliferation and resistance to treatment. Mechanisms of resistance have been found, on the one hand in elevated expression of the multidrug resistance protein 1 (MDR1) in breast cancer cells in response to to adriamycin, or on the other hand by neutralizing the effect of oxidative-stress promoting agents due to FOXO1-elicited upregulation of anti-oxidative enzymes (e.g., paclitaxel-resistant ovarian cancer) [177,178,179]. A further revealed cause of drug resistance was presented in renal cell carcinoma that was provoked by inhibitory drugs of the PI3K pathway. Subsequent AKT phosphorylation and activation causing renal tumor growth was mediated by upregulated FOXO activity and mTORC2 induction (via RICTOR expression) in an amplifying loop [287]. 

### 5.7. DAPK Upregulation

DAPK is a calcium/calmodulin-regulated protein kinase that is involved in the regulation of various cellular processes, such as caspase-independent cell death, apoptosis, and cytoskeletal activities [288]. It furthermore induces cell death signaling in response to inflammatory stimuli, such as interferon-gamma (INF-γ), tumor necrosis factor-alpha (TNF-α), and transforming growth factor-beta (TGF-β) where it interacts with different MAPKs, such as ERK [289]. DAPK has also been reported as an important regulator of autophagy involving several mechanisms [290]. By modulating the Vps34 class III PI3K complex, DAPK interferes with the process of autophagosome nucleation. This invovlves either DAPK-mediated phosphorylation of protein kinase D, which in turn phosphorylates Vps34, or the phosphorylation of Beclin-1, that also represents an essential component of the Vps34 complex [217,291]. Phosphorylation of of the BH3 domain of Beclin-1 by DAPK causes it to dissociate from anti-apoptotic BCL-2 family members such as Bcl-2 or Bcl-XL, and this event promotes the inhibition of the PI3K complex and stimulation of autophagy [218,251]. Moreover, independently from Beclin-1, DAPK has also been shown to activate autophagy via interaction with LC3 [252]. 

LBH589/panobinostat-mediated dephosphorylation of serin308 of DAPK affecting direct protein interactions was determined as the single cause of DAPK-induced autophagy in HCT116 colon cancer cells [147].

DAPK-based drug resistance against erlotin and cetuximab in cell lines of non-small cell lung cancer or head and neck squamous cell carcinoma was found to be caused by hypermethylation of the DAPK-encoding gene [180]; this could be reproduced by siRNA-mediated knockdown of *DAPK* while a gene transfers rescued resistance. Similarly, TSA re-sensitized cisplatin-resistant A549 cells to apoptosis by up-regulating *DAPK* [292]. Another study identified KLHL20-Cul3-ROC1 as an E3 ubiquitin ligase for DAPK-mediated degradation and therefore a regulatory mechanism that could explain resistance to INF-γ cell death signaling in cancer therapy [181].

### 5.8. NRF2 Upregulation

NRF2 is a transcriptional regulator of oxidative as well as xenobiotic stress (environmental toxicants or harmful chemicals)-induced cytoprotective genes, such as glutathione peroxidase, superoxide dismutase, and thioredoxin. Corresponding signaling is mediated by the NRF2-KEAP1 (kelch-like-associated protein 1)-Antioxidant Response Element (ARE) pathway. Autophagy has been implicated in the regulation of oxidative stress by undergoing the NRF2/KEAP1) and SQSTM1/p62 pathway [293,294,295]. SQSTM1/p62 represents an autophagy cargo adapter and substrate that can bind and guide KEAP1 to the autophagosome, thereby enabling its selective degradation [296,297]. This interaction furthermore releases NRF2 which subsequently translocates to the cell nucleus, where it binds to ARE sequences, which transcriptionally activates the expression of about 500 different genes, many of them possessing antioxidant effects [298]. In contrast to physiological conditions, where NRF2 is sequestered by KEAP1 and inactivated by proteasomal degradation, oxidative stress prolongs the extension of its half-life and transcriptional regulation involving, either upregulated SQSTM1/p62, or inhibited KEAP1 activity.

NRF2-signaling, mediated by microRNA-mediated regulation of mTOR, was also delineated as a novel regulator of HDACi-induced autophagy [146]. Thus, treatment of the hepatocyte-derived carcinoma lell line Huh-7, and of the primary gastric adenocarcinoma cell MGC-803, with TSA and SAHA upregulated NRF2 which subsequently increased expression the miRNA-129-3p by binding to its ARE1 site. MiRNA-129-3p in turn, induced autophagy and presumably also cell death by inhibiting mTOR, increasing the conversion of LC3-I to LC3-II, and downregulating SQSTM1/p63 expression. 

Comparable to other autophagy-associated genes, aberrant expression of NRF2, due to reduced KEAP1 activity, is correlated to chemoresistance by facilitating stress reduction [182,183,184]. In fact, NRF2-activated autophagy has been identified as an underlying mechanism. Consequently, a knock-down of NRF2 yielded increased sensitivity of tumor cells to anticancer agents; moreover, cancer patients with low NRF2 levels had better results following chemotherapeutic treatment [299,300]. Thus, the NRF2-miRNA-129-3p-mTOR signaling axis, that represents a sensor for chemical-induced oxidative stress, is important for the maintenance of cellular homeostasis; it has been potentially associated with drug resistance and might serve as a useful therapeutic target [301]. 

## 6. Concluding Remarks

Chemotherapeutic resistance has been considered a major challenge in cancer that also affects HDACi treatment [185,186,188]. While the development of isoform-selective HDAC-specific inhibitors are still in progress, many lines of evidence including experimental and clinical studies attest a promising effect for the use of HDACi as epigenetic drugs in cancer treatment; nevertheless, several obstacles related to solid malignancies have been encountered [302,303,304]. Furthermore, exact mechanistic insights related to the effector and resistance mechanisms of HDACi-induced cell death and the evaluation of molecular determinants, enabling a reliable indication of the treatment outcome, are still incomplete. This might be owned to the non-exhaustively explored, versatile mechanisms of action of HDACi, influenced by the class and dose of HDACi, and their combinatiorial effects with other drugs on the one hand. Nonetheless, reasons could also be found in cancer-cell specific effects and context-dependent molecular effects influenced by tumor-specific mutations or microenvironmental conditions (e.g., oxidative stress), on the other hand. 

Autophagy is a potential target for cancer therapy but is associated with an ambiguous role in diverse phases of tumorigenesis. Consistently, HDACi-mediated autophagy has been attributed with a critical role in HDACi resistance. Particularly, as the combination of inhibitors of autophagy with HDACi treatment has been established as an advanced therapeutic approach to re-sensitize cancer cells, the definition of decisive factors that direct autophagy towards a tumor suppressive or survival mode would be essential [47,187]. Comparable to apoptosis however, autophagy is governed by an intriguingly complex network of cellular signaling pathways. A focus of future studies should therefore pose the clarification of additional, non-elucidated molecular mechanisms of HDACi-mediated cell death and its determinants of resistance. The acquired information will allow the design of improved anti-tumor HDACi-composed drugs that include targeting of autophagy and even enable the selection of cancer therapy-responsive beneficiaries prior to treatment.

## Figures and Tables

**Table 1:** Mechanisms of HDACi-induced autophagy and potential mechanisms of resistance.

Autophagic Regulation	HDACi	Cancer Cell Type	Ref.	Mechanisms of Resistance	Ref.
mTOR Inhibition	SAHA	ESS-1	[49,153]	Constitutive PI3K-AKT signaling -AKToverexpression -PI3K mutations -Loss of PTEN, ARHI, LKB-1 -other upstream factors Beclin-1 deficiency Bcl-2 overexpression	
	SAHA	Glioblastoma	[136]		
	Butyrate, SAHA	HelaS3	[135,154]		
	SAHA, OSU-HAD, C42	HCC, Hep3B, HepG2	[139]		[155,156,157,158]
	SAHA	Jurkat T-cells	[137]		
	SAHA	Gliobastoma SC	[138]		
	Apicidin	Salivary MEC	[134,159]		[160,161,162,163]
	* MGCD0103	Primary CLL	[151,152]		
	* SAHA, TSA, VPA, MS-275, JQ2	DS-AMKL cells	[64]		
ROS Accumulation	SAHA	Jurkat T-cells	[137]	Increased levels of ROS scavengers/antioxidant enzymes (TRX)	[164,165]
	SAHA	CMLL	[58]		
	FK228	Gastric carcinoma	[165]		
	M-275	HCT116	[166]		
	VPA, SAHA	AML (Kasumi-1)	[62]		
	VPA, TSA	PaCa44, Panc1	[130]		
p53 Acetylation & Deficiency	Sirtinol	MCF-7	[131]	Overexpression/GOF of mutant p53 Decreased degradation of p53 (MDM2, HSP90)	[167,168,169,170,171]
	MHY2256	MCF-7	[132]		
	MHY2256	Endometrial	[133]		
	SAHA	ESS-1	[49]		
	VPA, TSA	PaCa44, Panc1	[130]		
p21 Upregulation	SAHA, H40	PC-3M, HL-60	[142]	Deregulated phosphorylation by Akt1 Deregulation by p53 (p53R2; cytoplasmic p21)	[172,173,174,175]
	MRJF4	PC3	[128]		
	Apicidin	Salivary MEC	[134,159]		
	VPA, TSA	PaCa44, Panc1	[130]		
NF-κB Hyper-acetylation	SAHA, MS-275	PC3	[141]	(Constitutive) NF-κB upregulation	[176]
FOXO1 Transcription	LBH589	HepG2, HCT116	[145]	MDR1 upregulation Anti-oxidative enzyme upregulation	[177,178,179]
DAPK Upregulation	SAHA, TSA, LBH589, JQ2	HCT116	[147]	DAPK hypermethylation & ubiquitination	[180,181]
NRF2 upregulation	SAHA, TSA	Huh-7, MGC-803	[146]	Aberrant NRF2 expression	[182,183,184]

* Suppression of autophagy due to mTOR activation; HDACi, histone deacetylase inhibitor; SAHA, suberoylanilide hydroxamic acid; VPA, valproic acid; TSA, trichostatin A; MGCD0103, mocetinostat; MS-275, etinostat; FK228, romidepsin; LBH589, panobinostat; SC, stem cells; MEC, Mucoepidermoid carcinoma; CLL, chronic lymphocytic leukemia;; DS-AMKL, down syndrome associated acute myeloid leukemia; AML, acute myeloid leukemia; MDM2, mouse double minute 2; TRX, thioredoxin; HSP90, heat shock protein 90.
